# Pathophysiology and Diagnosis of Drug-Induced Immune Thrombocytopenia

**DOI:** 10.3390/jcm9072212

**Published:** 2020-07-13

**Authors:** Caroline Vayne, Eve-Anne Guéry, Jérôme Rollin, Tatiana Baglo, Rachel Petermann, Yves Gruel

**Affiliations:** 1 EA 7501-Groupe Innovation et Ciblage Cellulaire (GICC), Université François Rabelais, CEDEX 01, 37032 Tours, France; caroline.vayne@univ-tours.fr (C.V.); jerome.rollin@univ-tours.fr (J.R.); 2 Laboratoire d’Hématologie-Hémostase, Hôpital Trousseau, CHRU Tours, CEDEX 09, 37044 Tours, France; eve-anne.guery@chu-tours.fr (E.-A.G.); tatianabag@yahoo.fr (T.B.); 3 Laboratoire d’Hématologie, CNHU de Cotonou, Cotonou 01 BP 386, Benin; 4 Département d’Immunologie plaquettaire, Institut National de la Transfusion Sanguine (INTS), 75015 Paris, France; rpetermann@ints.fr; 5 Equipe ETRES (Ethics, Research, Translations), Centre de Recherche des Cordeliers, UMRS 1138, INSERM, Sorbonne Université, Université de Paris, 75006 Paris, France

**Keywords:** platelets, thrombocytopenia, drugs, heparin-induced thrombocytopenia

## Abstract

Drug-induced immune thrombocytopenia (DITP) is a life-threatening clinical syndrome that is under-recognized and difficult to diagnose. Many drugs can cause immune-mediated thrombocytopenia, but the most commonly implicated are abciximab, carbamazepine, ceftriaxone, eptifibatide, heparin, ibuprofen, mirtazapine, oxaliplatin, penicillin, quinine, quinidine, rifampicin, suramin, tirofiban, trimethoprim-sulfamethoxazole, and vancomycin. Several different mechanisms have been identified in typical DITP, which is most commonly characterized by severe thrombocytopenia due to clearance and/or destruction of platelets sensitized by a drug-dependent antibody. Patients with typical DITP usually bleed when symptomatic, and biological confirmation of the diagnosis is often difficult because detection of drug-dependent antibodies (DDabs) in the patient’s serum or plasma is frequently not possible. This is in contrast to heparin-induced thrombocytopenia (HIT), which is a particular DITP caused in most cases by heparin-dependent antibodies specific for platelet factor 4, which can strongly activate platelets in vitro and in vivo, explaining why affected patients usually have thrombotic complications but do not bleed. In addition, laboratory tests are readily available to diagnose HIT, unlike the methods used to detect DDabs associated with other DITP that are mostly reserved for laboratories specialized in platelet immunology.

## 1. Introduction

Many drugs and components including herbal remedies, food, and nutritional supplements can cause thrombocytopenia by inhibiting platelet production and/or favoring their elimination or destruction from the peripheral blood [1]. Cytotoxic chemotherapies frequently suppress haematopoiesis overall, but some have a greater impact on megakaryocytopoiesis. On the other hand, peripheral drug-induced thrombocytopenia, characterized by increased clearance of platelets by mononuclear phagocytes, is most often mediated via an immunological mechanism implicating drug-dependent antibodies, which may also induce direct platelet destruction [2,3,4,5]. This latter entity called drug-induced immune thrombocytopenia (DITP) is not exceptional, but often is a real diagnostic challenge. Indeed, most patients with DITP have multiple comorbidities and other potential causes of thrombocytopenia. The objective of this review is to present and summarize the different pathophysiological mechanisms and drugs involved in DITP, and some practical key points useful for their diagnosis are also discussed. 

## 2. Drugs and Mechanisms Involved in Drug-Induced Immune Thrombocytopenia

Several studies in the past years have allowed identification of many drugs responsible in the occurrence of DITP [6,7,8,9]. A reliable Web resource called “Platelets on the Web” (https://www.ouhsc.edu/platelets/ditp.html) was developed by Dr James N. George for collecting patient reports of immune mediated thrombocytopenia (last update in 2018), and currently lists more than 300 drugs with which at least one confirmed or suspected case of DITP has been described.

Quinine was the first drug to be identified as causing immune-mediated thrombocytopenia over 100 years ago, but the incidence of DITP with this drug is rare, only of 26 cases per million patients treated [10]. Heparin-induced thrombocytopenia (HIT) is more frequent, affecting more than 1% of treated patients in certain clinical situations, and is distinguished from other DITP because it is associated in nearly half of all cases with thrombotic complications, and rarely with severe bleedings [11,12].

The pathophysiological mechanisms underlying DITP are quite variable (Table 1), differ according to the molecules involved [1,4], and are discussed below.

### 2.1. Thrombocytopenia Induced by Hapten-Dependent Antibodies

The first observations of DITP were initially attributed to hapten-dependent antibodies. Haptens are molecules that are too small to be immunogenic alone, but after their covalent binding to macromolecules such as proteins, drug-specific antibodies can be synthesized and target platelets. This mechanism has been reported with cephalosporin drugs or penicillins, which may bind via their β-lactam ring to the membrane of red blood cells and induce immune haemolytic anaemia. Penicillin or derivatives may also bind covalently platelet glycoproteins and induce antibody response with subsequent immune thrombocytopenia [13,14,15]. However, this process is rare, and in patients treated by penicillin derivatives, the antibodies are more frequently acting like those involved in “Quinine-type” DITP (see Section 2.2).

Piperacillin has also been implicated in the occurrence of immune thrombocytopenia [37], but with a link not fully demonstrated. Cephalosporins, more particularly ceftriaxone, were also reported to be responsible for DITP, with antibodies recognizing GPIIb/IIIa or GPIb/IX, especially the GPIX subunit [16].

### 2.2. “Quinine-Type” Drug-Induced Immune Thrombocytopenia 

DITP induced by “drug-dependent” antibodies (DDabs) are classically due to antibodies inducing platelet destruction by the reticuloendothelial system only in the presence of the drug. Quinine, an anti-malarial drug also frequently prescribed for the treatment of leg cramps, is the first drug that was recognized to be involved in this group of DITP. However, “quinine-type” drug-dependent antibodies have also been identified in patients with thrombocytopenia induced by variable other drugs such as non-steroidal anti-inflammatory drugs, antibiotics (ceftriaxone, piperacillin, vancomycin, rifampicin, trimethoprim/sulfamethoxazole, and teicoplanin), and anticonvulsants (phenytoin and carbamazepine). Actually, more than 100 different medications have been implicated in this group of DITP [3,5,17,18], but vancomycin is today one of the most frequently involved in clinical practice [38].

”Quinine-type” antibodies typically recognize restricted binding sites expressed by GPIb/IX and GPIIb/IIIa complexes of the platelet membrane [39,40,41,42]. Antibodies induced by antibiotics such as vancomycin [41], sulfamethoxazole [43], or teicoplanin [44] preferentially bind GPIIb/IIIa. Quinine-type” antibodies may interact either with GPIIb or GPIIIa alone, but also with the intact integrin. This is also true for antibodies that bind to GPIb/IX complex. In this regard, the GPIX subunit appears to be the preferred target of rifampicin-dependent antibodies [45]. Moreover, the Arg110 residue in the GPIX subunit has been identified as playing a critical role in the antigenic site [46]. Recently, it was demonstrated that quinine is retained specifically by human GPIX, with a binding site involving residues 110–115 [47].

**Figure 1:**
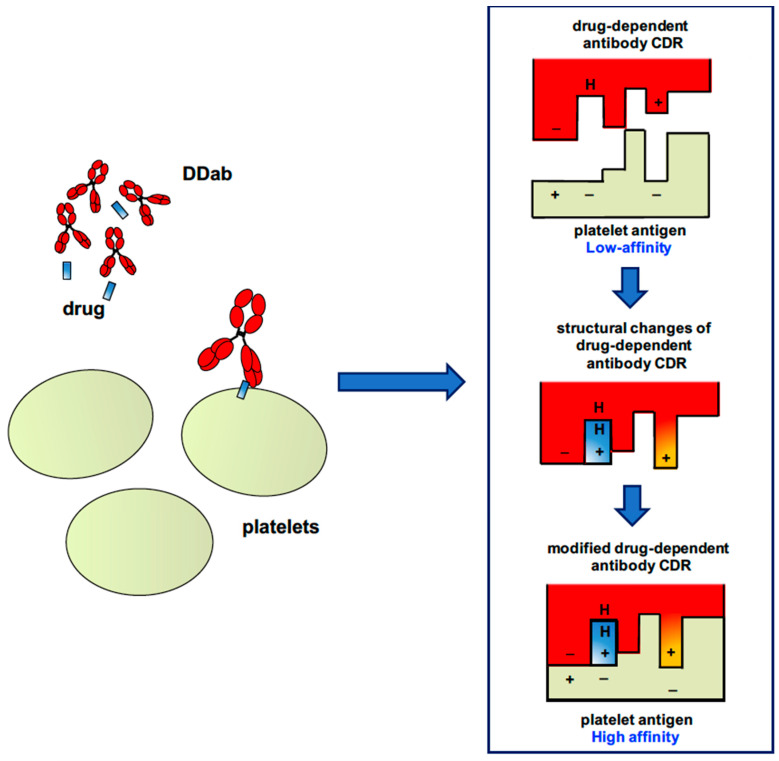
Schematic representation of the binding of a “quinine-type” drug-dependent antibody to a platelet glycoprotein. CDR, complementary-determining region; DDab, drug-dependent antibody.

Unlike the hapten-dependent mechanism, there is no covalent interaction between the drug and the antigenic target, and quinine-dependent antibodies have a low affinity for platelet glycoproteins (GPs). However, importantly, the drug strongly increases antibody binding on platelets. How the sensitized drug promotes the binding of quinine-type antibodies to the membrane has remained unknown for many years. However, recent studies have proposed a mechanism that involves the interaction of the drug with the complementary-determining region (CDR) of the antibody, whose configuration is then modified with a subsequent increased antibody affinity for a specific epitope expressed by a platelet glycoprotein. As illustrated in Figure 1, the drug is likely trapped at the antigen–antibody interface in a resulting tri-molecular complex [4,48].

Recent studies using different monoclonal antibodies that mimic antibodies of patients with “quinine-type” DITP then supported this model. Bougie et al. confirmed that the first step was the interaction of the drug with antibody, inducing structural changes, which then strongly increase its affinity and specificity for its target epitope [49]. In addition, Zhu et al. from the same team also showed how quinine could remodel the paratope of the fragment antigen-binding (Fab) antibody by changing the conformation of CDR loops [48]. These findings suggest that synthesis of “quinine-type” antibodies is triggered, whatever the drug involved, by conformational changes of B-cell receptor (BCR), which allow it to acquire high specificity to an epitope expressed by a platelet glycoprotein.

Antibody-sensitized platelets are usually rapidly eliminated but some quinine-type antibodies may also bind megakaryocytes and impair platelet production by decreasing the proplatelet production capacity, this effect possibly prolonging thrombocytopenia in some patients [50].

### 2.3. Immune Thrombocytopenia Induced by GPIIb/IIIa Inhibitors

#### 2.3.1. Thrombocytopenia Induced by Abciximab

Abciximab is a chimeric (human/mouse) anti-GPIIb/IIIa Fab fragment that blocks the binding of fibrinogen to platelet GPIIb/IIIa [51], and it was mainly used to prevent ischemic cardiac complications in acute coronary syndromes in patients undergoing percutaneous coronary intervention. The incidence of thrombocytopenia after injection of abciximab is 1 to 2% at the first exposure [19], but it is more frequent when the drug is later administered. Approximately 10–15% of patients receiving abciximab twice in 30 days may develop thrombocytopenia [20]. Acute thrombocytopenia occurs few hours after starting treatment, with sometimes fever, dyspnea, hypotension, and rarely anaphylactic shock. Usually, bleeding manifestations are transient and not severe, but life-threatening intracranial haemorrhages have been reported. Thrombocytopenia is usually profound with platelet counts between 1 and 25 × 10^9^/L, and due to IgG/IgM antibodies specific to the platelet-bound drug. Similar antibodies can also be found in healthy subjects, but their specificity is likely different. Pathogenic antibodies, i.e., dangerous and potentially inducing acute thrombocytopenia, recognize murine sequences present in the abciximab molecule and necessary for its binding to GPIIb/IIIa, whereas non-pathogenic antibodies (not associated with low platelet counts) are specific for the papain-cleaving site of abciximab Fab fragment [21]. In some patients, antibodies that bind to conformational epitopes induced in GPIIb/IIIa by abciximab can also provoke thrombocytopenia [52].

Most patients recover within a few days (in general five days), but sometimes low platelet count may persist several weeks [53]. On the other hand, delayed thrombocytopenia after abciximab may also be observed in few patients, with a decrease in platelet count 5 to 10 days after treatment. This is explained by the fact that abciximab remains present on circulating platelets for up to two weeks, because the drug can move from one cell to another [54]. Therefore, abciximab-induced delayed thrombocytopenia may be caused by newly synthesized antibodies to the drug remaining on the platelet surface for an extended period of time. This form usually occurs after hospital discharge, may be severe, with a diagnosis sometimes delayed, especially in patients treated by other antiplatelet agents such as aspirin or P2Y12 inhibitors that may also favor the occurrence of severe bleeding. It is therefore recommended that patients be sensitized to carefully look for petechiae or muco-cutaneous bleedings after leaving the hospital [21].

#### 2.3.2. Immune Thrombocytopenia Induced by Ligand-Mimetic Fibrinogen-Receptor Antagonists

Ligand mimetic fibrinogen-receptor antagonists are therapeutic agents mimicking the arginine-glycine-aspartic acid (RGD) sequence recognized by specific sites of the platelet GPIIb/IIIa complex. Therefore, they competitively inhibit fibrinogen-GPIIb/IIIa interactions and the formation of platelet aggregates. The two compounds used to prevent thrombotic complications associated with percutaneous transluminal coronary angioplasty are tirofiban and eptifibatide, a cyclic hexapeptide. About 0.1 to 0.5% of patients treated for the first time with these drugs may develop thrombocytopenia [22]. This adverse event typically occurs within the first 24 h of treatment, and fever, chills, and hypotension can be observed in some cases. However, most patients recover without severe bleeding and further problems in a few days. 

Platelet destruction in affected patients is due to antibodies recognizing a neoepitope or a ligand induced binding site (LIBS) expressed by GPIIb/IIIa whose conformation has been altered after drug binding [55]. Eptifibatide-dependent antibodies do not bind to GPIIb/IIIa in the presence of tirofiban, and vice versa, with some exceptions. These antibodies may also be natural, without prior exposure to the drug, explaining the rare cases of thrombocytopenia observed on first exposure a few hours after administration [56,57]. 

Thrombocytopenia is usually severe between 1 and 25 × 10^9^/L, requiring in some cases platelet transfusions and intravenous (IV) immunoglobulins. Platelet count recovery most often occurs within 2–3 days, but can be delayed in case of direct inhibitory effect of antibodies on megakaryocytes [58]. Thrombocytopenia may rarely be associated with paradoxical thrombotic events [59,60,61], which may be related to the ability of some antibodies to activate platelets after cross-linking FcγRIIa receptors [61], in a way similar to the process involved in heparin-induced thrombocytopenia (see Section 2.4.2).

### 2.4. Drug-Induced Immune Thrombocytopenia from Other Causes

#### 2.4.1. Thrombocytopenia Induced by Platelet-Specific Auto-Antibodies

Some drugs are capable of inducing the production of autoantibodies that cause, in the absence of the sensitizing molecule, platelet destruction in a way similar to that involved in immunological thrombocytopenic purpura. This process has been found in 1% to 3% of patients treated for rheumatoid arthritis with gold salts [23,24] that are no longer used in medical practice, and also been demonstrated with procainamide [25] and L-dopa [26]. Auto-antibodies induced by gold salts were specific for platelet glycoprotein V and not found in non-thrombocytopenic treated patients [62]. 

Several humanized monoclonal antibodies, approved these last years for the treatment of malignant and non-malignant disorders, have also been identified as potentially inducing the formation of autoantibodies targeting platelets. A moderately low platelet count is relatively common upon exposure to these agents, but more severe thrombocytopenia has also been reported in a few patients. The drugs involved are efalizumab (anti-CD11a) [63,64], adalimumab [65] and infliximab (anti-TNF, tumor necrosis factor) [28,29], bevacizumab (anti-VEGF) [27,66], rituximab (anti-CD20) [30,31], natalizumab (anti-α_4_β_1_-integrin) [32], and immune checkpoint antibodies to program cell death receptor-1 (PD-1) or cytoxic T-lymphocyte antigen 4 (CTLA-4), such as nivolumab, pembrolizumab, or ipilimumab [33,67]. Although the clinical evolution in most cases suggests that antibodies are involved in platelet destruction, their presence has never been firmly demonstrated. Platelet count usually falls within a few days of treatment, sometimes with bleedings, and recover rapidly after the drug withdrawal. Moreover, recurrence of thrombocytopenia is inconstant after re-exposure [28,64].

#### 2.4.2. Thrombocytopenia Induced by Immune Complexes: Heparin-Induced Thrombocytopenia 

In this group, the model disorder is heparin-induced thrombocytopenia (HIT), which clearly differs from other DITP since it is associated with venous or arterial thrombotic complications (50% of cases) and rarely haemorrhagic events [12] (Table 2).

HIT occurs in 1 to 3% of patients treated with unfractionated heparin (UFH), with a lower incidence in patients receiving a low molecular weight heparin (LMWH), and is difficult to predict, depending on the clinical context. The decrease in platelet count is classically greater than 40% and occurs typically between 5 and 10 days after the start of treatment. This iatrogenic complication is due to an atypical immune response leading to the synthesis of pathogenic antibodies of IgG isotype specifically directed in most patients against platelet factor 4 (PF4) modified by heparin (PF4/H). Indeed, heparin and other sulfated polyanions are capable of rendering PF4 highly immunogenic by promoting conformational changes on its surface [68] and the aggregation of several tetramers to form ultra-large complexes (ULC) [69]. The size of the polyanions and their degree of sulfation (number of negative charges per sugar) are two parameters that strongly influence the formation of large antigenic complexes [70].

This explains why unfractionated heparin, which contains large amounts of chains of more than 12 sugars and has a high degree of sulfation, is particularly prone to induce these changes and the development of an immune response against modified PF4. 

This immune response is complex and atypical in several respects: HIT is very common compared to other DITPs, it is transient with no immunological memory [71], and there is no isotype switch with concomitant synthesis of IgG/IgA/IgM antibodies, which progressively disappear within three to six months following heparin discontinuation [72]. 

A pre-immunization step related to previous bacterial infections may explain the rapid synthesis of IgG, which begins after 4–5 days of exposure to heparin [73]. In addition, inflammation appears to play an important role in the loss of immune tolerance to PF4/H complexes, as suggested by reduced levels in patients with HIT of anti-inflammatory cytokines, such as interleukine 10 (IL-10) or transforming growth factor β (TGF-β) [74]. Data from murine models have also indicated that marginal area B cells, generally involved in T cell independent humoral responses, are essential in the synthesis of anti-PF4/H antibodies [75], but several teams have also proposed a role for helper T cells in this process [76]. Recently, a role of complement and endogenous polyreactive IgM in the immune response leading to HIT has also been demonstrated [77]. 

In addition to the use of heparin, other factors may promote the development of antibodies to PF4, including clinical conditions associated with high platelet activation and increased PF4 release (e.g., cardiac surgery with cardiopulmonary bypass, and orthopedic surgery) [78].

Antibodies specific to PF4 are very heterogeneous in terms of class, subclass, affinity, and specificity. The majority of patients develop antibodies directed against conformational epitopes induced by the interaction of PF4 with heparin or other glycosaminoglycans [79]. However, a few patients may also develop atypical antibodies capable of binding PF4 alone [80]. In this respect, Nguyen et al. recently showed that such antibodies were present in patients with atypical (so-called autoimmune) HIT, characterized by an unusual clinical presentation such as spontaneous HIT, and delayed or persistent HIT despite withdrawal of heparin [81]. Antibodies present in patients with autoimmune HIT activate platelets without heparin, and although their role is not yet fully understood, they may promote binding of anti-PF4/H antibodies to PF4 alone. All these characteristics explain why this particular bio-clinical HIT is now considered as an autoimmune disease [82].

The pathogenicity of HIT antibodies is mainly due to IgG that activate platelets after binding of their crystallizable fragment (Fc) to FcγRIIa receptors [83,84]. This activation induces the release of PF4 from platelet alpha granules and generates microparticles rich in phosphatidylserine (PS), this providing a procoagulant surface favoring the generation of thrombin [85,86]. HIT antibodies also induce FcγRIIA-dependent monocyte activation that leads to synthesis of tissue factor (TF), the main trigger of coagulation [87]. The release of procoagulant microparticles loaded with TF by activated monocytes, allowing explosive thrombin generation, would also contribute to enhance platelet activation and thrombus formation [88]. In addition, recent studies demonstrated that generation of Neutrophil Extracellular Traps (NETs) from neutrophils induced by HIT antibodies was critical for the development of thrombosis [89,90]. On the other hand, von Willebrand factor (vWF), a multimeric protein released from activated endothelial cells, also contributes to antibody binding to the endothelium by forming antigenic complexes with PF4 [91]. These vWF/PF4/antibody complexes would recruit the platelets via the Fc fragment of IgG, thus further contributing to the development of thrombi. 

HIT is relatively common in cardiac surgery patients who are exposed to high doses of UFH during cardiopulmonary bypass (CPB). However, in this particular clinical setting, patients may also develop specific antibodies to protamine [34,35,36], another positively charged protein, which like PF4 has a high affinity for heparin. Although these antibodies can form immune complexes and induce thrombocytopenia in some patients, their pathogenic effect is weaker than that of typical HIT antibodies to PF4/heparin complexes [92].

### 2.5. Unresolved Questions on the Pathogenesis of Drug-Induced Immune Thrombocytopenia

The decrease in platelet count is usually rapid and profound in typical DITP, while thrombocytopenia is rather moderate in most patients with HIT, and associated with cell activation. These differences likely result from the involvement in DITP of various mechanisms in the platelet clearance process, with potential roles of complement and FcγRs, which are not fully identified, especially in DITP. In addition, an effect of antibodies on the platelet production has also been showed in a few cases.

#### 2.5.1. Role of Complement in Drug-Induced Immune Thrombocytopenia

Complement activation may also contribute to platelet destruction in DITP, as initially suggested in 1958 by Schulman who had detected quinidine-induced DDabs in patients with DITP using a complement fixation assay [93]. Kiefel then showed that quinidine and rifampicin-dependent antibodies induced the binding of large amounts of C3d and C5b-C9 components to the platelet surface [94]. Furthermore, the role of complement in DITP was also supported by the fact that antibodies induced by vancomycin, quinine, or fluoroquinolones [41,95,96], are often IgM, a class of immunoglobulins which is particularly prone to activate the complement pathway due to its pentameric organization. 

#### 2.5.2. Role of Fcγ Receptors in Drug-Induced Immune Thrombocytopenia

In humans, Fcγ receptors (FcγRs), i.e., FcγRI (CD64), FcγRIIA (CD32A), FcγRIIB (CD32B), FcγRIIC (CD32C), FcγRIIIA (CD16A), and FcγRIIIB (CD16B), are mainly expressed by haematopoietic cells and important for the promotion and regulation of immune and inflammatory responses [97]. Today, it is well established that the pathogenicity of HIT antibodies mainly depends on cell activation resulting from the interaction of anti-PF4/heparin IgG with Fcγ receptors, and particularly FcγRIIA [88,90,98]. In addition, two different teams have demonstrated that the H131R polymorphism located in the IgG binding region of FcγRIIA influences the risk of thromboembolic complications in HIT [98,99], and this association has recently been confirmed by analyzing a large prospective cohort of patients with definite HIT [100]. 

Apart from being critical in cell activation induced by HIT IgG antibodies, FcγRs are also important in the clearance of opsonised platelets by macrophages and dendritic cells. The involvement of FcγRIIA in this process has been demonstrated in a murine model expressing human FcγRIIA [101], but the role of FcγRIIIA is also likely critical. In this regard, one polymorphism (FcγRIIIA V158F) strongly influences the affinity of human IgG1 and IgG3 to the receptor, i.e., this affinity is higher to the V allotype [97]. Interestingly, IgG antibodies to PF4/heparin are predominantly IgG1 and IgG3 [80,102], and we found that the risk of HIT was stronger in patients with high levels of antibodies and homozygotes for the FcγRIIIA 158V allele [103], suggesting that FcγRIIIA-mediated platelet clearance also contributes to decreasing the platelet number during HIT.

On the other hand, the role of FcγRs in other DITP has not been clearly established, although, as for other immune thrombocytopenia, they should be involved in the clearance by macrophages and dendritic cells of platelets opsonised by IgG DDabs, [101,104]. In support to this hypothesis, studies performed in patients with DITP, or using a murine model, have demonstrated that intravenous immunoglobulins, which are interfering with FcγRs, are able to inhibit platelet clearance induced by different DDabs, including quinine-dependent and HIT antibodies [105,106,107].

#### 2.5.3. Effect of Drug-Dependent Antibodies on Platelet Production

The suppression of megacaryocytopoiesis is readily attributable to drugs that induce global myelosuppression, such as chemotherapies, or other drugs that selectively affect platelet production by megakaryocytes (colchicine, tolbutamide, and thiazidic diuretics) [108]. On the other hand, the inhibitory effect of anti-platelet autoantibodies associated with immune thrombocytopenia (ITP) on platelet production has also been clearly documented [109,110], explaining the benefits of treating patients with chronic ITP with thrombopoietin receptor agonists. 

On the other hand, the potential role of DDabs in suppressing platelet production has been rarely discussed. In 1983, Murphy et al. reported a case of penicillin-induced neutropenia and thrombocytopenia [14]. The hypocellularity of the bone marrow at the time of diagnosis, its normalization after drug withdrawal, and the demonstration of complement-fixing IgG antibodies reacting with patient’s neutrophils and platelets in the presence of the drug, supported the existence for the authors of antibody-mediated suppression of penicillin-coated precursor cells, although no studies have confirmed this hypothesis.

Megakaryocytes express glycoproteins and other platelet antigens, making them a potential target of DDabs, as supported by data showing that monoclonal and human antibodies to GPIb and GPIIb/IIIa are able of inhibiting megakaryocytopoïsesis in vitro [109]. Moreover, Greinacher et al. demonstrated that eptifibatide, a GPIIbIIIa inhibitor, induced the development of auto-antibodies that bind to megakaryocytes, decrease their viability, and can cause prolonged thrombocytopenia [58]. Persistent thrombocytopenia in patients with quinine-induced DITP has also been attributed to anti-GPIb/IX antibodies that bind megakaryocytes, induce their apoptosis, affect their differentiation, and markedly decrease proplatelet production [50]. The mechanisms underlying alteration of megakaryocytes by anti-platelet antibodies are not clearly understood, but Perdomo et al. suggested that anti-GPIb/IX antibodies may induce the production of reactive oxygen species that activate caspase-3 and promote cell apoptosis. These antibodies could also destabilize the interactions between GPIb and intra-cellular cytoskeleton, which is crucial for thrombopoiesis [111]. 

Inhibition of platelet production by DDabs may also help to explain why laboratory confirmation of the diagnosis of DITP may be difficult when trying to detect platelet-bound antibodies. For this reason, the use of megakaryocytes as test cells has recently been suggested to study the impact of DDabs on proplatelet production [112].

## 3. How to Diagnose Drug-Induced Immune Thrombocytopenia

**Figure 2:**
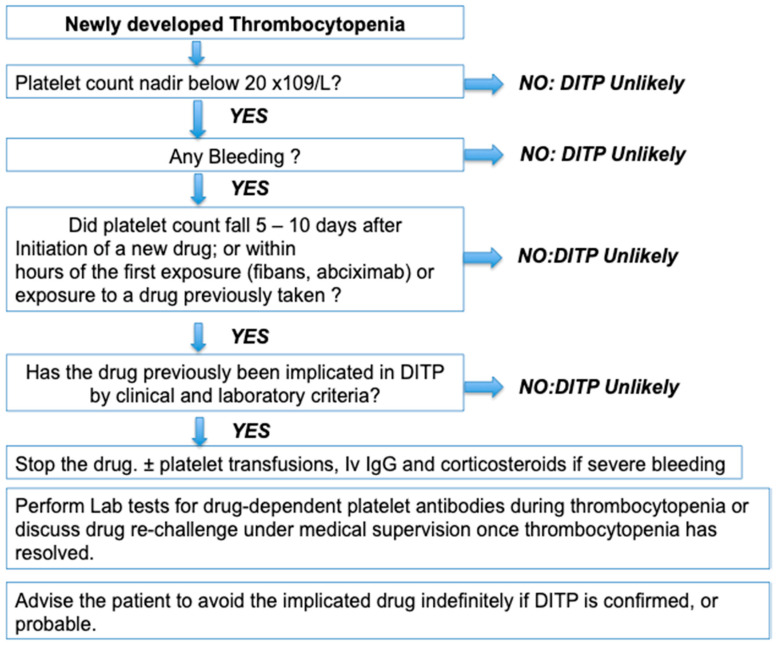
Practical approach for the diagnosis of DITP, adapted from Arnold et al. 2013 [5]. DITP: drug-induced immune thrombocytopenia; Iv Ig: Intravenous immunoglobulins.

DITP is a severe clinical syndrome often responsible for major thrombocytopenia, less than 20 × 10^9^/L, and may be associated with severe bleeding. Most symptomatic patients develop mucosal bleedings (purpura or epistaxis), but rarely, more serious clinical complications, such as intra cranial or intra pulmonary haemorrhages, which can compromise the patient’s vital prognosis [113] can also be observed. Thrombocytopenia usually occurs about 1 week after exposure, but in some cases within hours of taking for drugs previously administered occasionally or repeatedly. In practice, the diagnosis of DITP is difficult, mainly suspected on clinical criteria (Figure 2), and laboratory tests, which demonstrate the presence of drug-dependent platelet antibodies, are the only methods that can demonstrate, in some cases, the responsibility of specific drugs in the pathological process leading to thrombocytopenia. 

### 3.1. Clinical Features

Careful examination of affected patients is essential, looking for purpura-type bleedings, mucocutaneous signs (petechiae, ecchymosis), and sometimes digestive, genital, urinary or intracranial haemorrhages. The intake of foods, nutritional supplements, plants or beverages that may contain quinine must also be part of the questionnaire. Patients may also present with fever or nausea, but these are non-specific clinical signs since also found in sepsis, another possible cause of thrombocytopenia making the diagnosis of DITP sometimes difficult [114]. In addition, no bleeding is associated with thrombocytopenia in some patients. The diagnosis of DITP has to be suspected in all patients who develop severe thrombocytopenia usually after 5 to 7 days of starting one of the drugs previously defined as implying in DITP, or within hours of receiving other treatments such as abciximab, or fibrinogen receptor antagonists. 

In patients treated by several drugs including heparin, it is important to differentiate HIT from other DITP. While bleeding symptoms are frequent in other drug-induced thrombocytopenias, HIT is associated with a high risk of thrombosis and is therefore treated differently. Apart from these clinical differences, thrombocytopenia usually remains moderate in HIT with median values at nadir of 60 × 10^9^/L versus 10 × 10^9^/L in DITP. Arnold et al. have proposed an approach in 2013 (Figure 2) that allows assessing the possibility of DITP based on 4 criteria: (1) the severity of thrombocytopenia (2) the clinical signs (3) the time to onset (4) the use of drugs already identified as responsible for DITP (with clinical and laboratory tests) [5]. However, the diagnosis remains often tricky because many patients have associated co-morbidities and other possible causes of thrombocytopenia. One of the arguments in favour of the suspected drug may be the normalisation of the platelet count after stopping the drug or relapsing on re-exposure. But, withdrawal of the suspected drug can be challenging, as well as re-introduction testing is risky to perform, exposing the patient to recurrent thrombocytopenia and bleeding, and should therefore be conducted at a low dose, and under medical supervision. 

Ideally, antibodies bound to platelets in the presence of the drug should be detected by laboratory tests. However, the tests available today are only specialized techniques lacking standardization and performed in a few laboratories.

### 3.2. Laboratory Assays for the Diagnosis of Drug Immune Thrombocytopenia Are Poorly Standardized

In order to confirm the diagnosis of DITP, laboratory assays must fulfill four criteria: (1) the drug or one of its metabolites is required for the reaction observed in vitro; (2) a specific immunoglobulin binding is demonstrated; (3) platelets are the target of this binding; and (4) at least two laboratories must independently obtain the same biological results in favor of the diagnosis [115]. 

If all four criteria are fulfilled, the diagnosis of DITP is certain, but, if antibodies are found in only one laboratory, it is still probable. In other cases, the diagnosis of DITP remains uncertain. Based on this approach, from a list of 153 molecules, laboratory tests allowed for establishing a definite causal relationship with immune thrombocytopenia for 16 drugs (quinine, quinidine, trimethoprim/sulfamethoxazole, vancomycin, penicillin, rifampicin, carbamazepine, ceftriaxone, ibuprofen, mirtazapine, oxaliplatin, suramin, abciximab, tirofiban, eptifibatide, heparin) [9]. For 20 other drugs, their involvement was only likely, with positive laboratory tests in a single laboratory.

Recommendations were formulated by the International Society on Thrombosis and Haemostasis (ISTH) in 2015 [115] for the standardization of tests to be performed with the drugs most frequently involved in DITP, namely quinine, vancomycin, trimethoprim-sulfamethoxazole, and piperacillin/tazobactam. Among the possible techniques, two are considered preferable, flow cytometry and enzyme-immunoassays (ELISA). These recommendations are summarized in Table 3. Whatever the method used, it is recommended to test a drug concentration of 1 mg/mL in the patient’s serum or plasma and the washing or incubation buffers. However, the use of too high of a concentration may result in false positive results. This supports that therapeutic concentrations may be preferred for several drugs when performing the assay. However, the solubility of some drugs can also be problematic. 

For example, trimethoprim/sulfamethoxazole must be dissolved in aqueous solutions at neutral pH, but the use of a buffer with 5% bovine serum albumin is helpful for increasing the solubility of sulfamethoxazole [43]. 

Regardless of the technique used, it is essential to systematically include two controls, one positive and one negative. The ideal positive control is a patient’s serum containing antibodies specific to the incriminated drug, but in practice this is not often feasible and testing an anti HPA-1a is easier and allows for ensuring that the technique can detect antibodies bound to platelets. The ideal negative control is the serum of a patient who has received the drug but without developing thrombocytopenia. However, in practice, the serum of a healthy subject, easier to collect, can also be used. In addition, it is essential to test the patient’s serum in the absence of the drug to ensure that the antibody detected is drug-dependent. 

**Figure 3:**
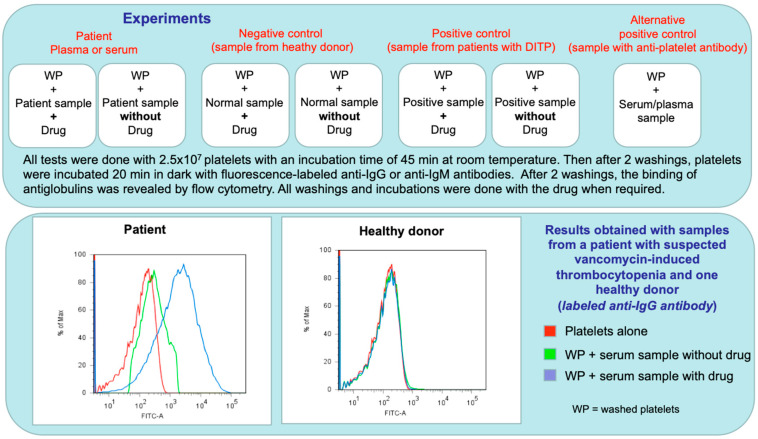
Example of flow cytometry analysis applied for the diagnosis of DITP due to vancomycin. Results obtained are shown in the lower panel. The procedure used was adapted from Curtis et al. [43]. Abbreviations: DITP: drug-induced immune thrombocytopenia; WP: washed platelets.

The test is considered as positive when significant antibody binding to platelets is demonstrated with the patient sample only in the presence of the suspected drug. An example of the procedure applied and results obtained with the serum of a patient with DITP while treated by vancomycin are shown in Figure 3. Results are usually expressed as a ratio of fluorescence intensities or optical densities measured with and without the drug and the ISTH subcommittee of standardisation recommended a positive cut off value of 1.5 [115]. However, it is preferable that each laboratory tests serum samples from 10 to 20 healthy subjects to define its own cut-off values. Importantly, the available laboratory tests have good specificity but poor sensitivity and therefore a negative result does not rule out DITP definitively. Several hypotheses can explain the poor sensitivity of the tests, but a common explanation is that DITP may be related to a metabolite and not to the drug itself. On the other hand, low solubility of the drug may interfere with testing. Finally, the antibody titer may be too low to be detectable in a sample collected too late.

All these difficulties and uncertainties explain that only specialized laboratories in platelet immunology should preferentially perform laboratory assays for searching drug-dependent antibodies, other than those involved in heparin-induced thrombocytopenia. 

### 3.3. The Diagnosis of Heparin-Induced Thrombocytopenia Is Easier to Confirm 

Since HIT is due in most patients to antibodies directed against PF4/heparin complexes, the laboratory diagnosis of this particular DITP is easier, and discussed in detail in a specific article of this journal [116].

Briefly, diagnosis of HIT involves several steps [11,117]. First of all, a clinical pre-test score (4Ts score) is frequently used to guide laboratory assays and allows for defining three levels of probability of HIT: low, intermediate and high, taking into account the severity of thrombocytopenia, the time to onset, the existence of thrombosis and other causes of thrombocytopenia. When the probability of HIT is intermediate or high, laboratory tests are then usually performed. Enzyme immunoassays are very sensitive to detect anti-PF4/H antibodies and frequently performed as first-line tests. These assays have a very good negative predictive value, close to 100%, but are not very specific [118]. Therefore, if positive, they should be combined with a functional test to demonstrate the ability of antibodies to induce heparin-dependent platelet activation [117,119]. Among the functional tests used in specialized laboratories, radiolabelled serotonin release assay (SRA) and heparin induced platelet activation assay (HIPA), which both use washed platelets from healthy subjects, are considered as the reference assays with a sensitivity and specificity of more than 95%. However, platelet aggregation tests, which are less sensitive, can also be performed. More recently, platelet activation assays performed with whole blood i.e., heparin induced multiple electrode aggregometry (HIMEA) and flow cytometry-based assays, have been proposed [120], but these methods have not yet been fully validated [121]. A decision algorithm for the clinico-pathological approach in any case of suspected HIT has to be used, but with a specific approach for patients after cardiac surgery for whom the 4T score is not easy to apply and less reliable [122].

In conclusion, thrombocytopenia is a frequent adverse event associated with the use of many different drugs, but the responsibility of antibodies in triggering platelet destruction and/or premature elimination is often difficult to demonstrate in most patients with typical DITP. In contrast, HIT is more frequent and easier to diagnose, since assays are widely available, and sensitive enough in detecting heparin-dependent antibodies.

## Figures and Tables

**Table 1:** Mechanisms involved in different types of drug-induced immune thrombocytopenia (DITP).

Type	Mechanism	Examples	References
**Hapten-induced antibody**	Drug binds to platelet membrane and promotes antibody response	Penicillin and derivatives, cephalosporins	[13,14,15,16]
**“Quinine-type” antibody**	Drug binds to antibody Fab and/or membrane glycoprotein (GP), thereby enhancing antibody affinity and binding to platelet GP	Quinidine, quinine, antibiotics (vancomycin, rifampicin, sulfamethoxazol), anticonvulsants	[3,4,5,17,18]
**Drug-specific antibody**	Antibody recognizes the monoclonal antibody bound to its target	abciximab	[19,20,21]
**Fibrinogen receptor antagonist-dependent antibody**	Drug binds to GPIIb/IIIa inducing conformational changes, then recognized by antibody	tirofiban, eptifibatide	[22]
**Autoantibody induction**	Drug induces formation of autoantibody that binds alone to platelet GP	procainamide, gold salts, L-dopa, and likely several therapeutic monoclonal antibodies	[23,24,25,26,27,28,29,30,31,32,33]
**Immune complexes**	Drug binds to PF4 inducing antibodies that activate platelets via FcγRIIa receptors	heparin, protamine	[11,12,34,35,36]

DITP: drug-induced immune thrombocytopenia; PF4: platelet factor 4; GPIIb/IIIa: glycoprotein IIb/IIIa.

**Table 2:** Main differences between heparin-induced thrombocytopenia (HIT) and other DITPs.

	HIT	Other DITPs
**frequency**	frequent	rare
**main mechanism of thrombocytopenia**	activation	consumption/destruction
**contribution of other cell types**	yes: leukocytes, endothelial cells	no
**time to occurrence after drug initiation**	mostly: 5–10 days	few hours to few days
**depth of thrombocytopenia**	moderate: nadir close to 50 × 10^9^/L in most cases	severe: nadir < 10–20 × 10^9^/L in most cases
**clinical manifestations**	thrombosis in 30-50% of cases; bleeding in < 10% of patients, in case of DIC	bleeding
**diagnosis**	affordable: well-established diagnostic approach, first-line tests: immunoassays, confirmation tests: functional assays	difficult: few assays available (immunoassays or flow cytometry-based assays) of unknown sensitivity, and restricted to specialized laboratories
**recurrence on re-exposure to the drug**	not systematically	very likely

HIT: heparin-induced thrombocytopenia; DIC: disseminated intravascular coagulation; DITPs: drug-induced immune thrombocytopenia.

**Table 3:** Laboratory testing for DITP: summary of recommendations from the SSC of the ISTH [115].

I. Sample Collection
**Timing**: - preferentially during the acute episode of thrombocytopenia - at least on a sample collected up to 3 weeks after the acute event **Anticoagulant**: - clotted serum or citrated plasma; avoid EDTA
**II. Preparation of Test Platelets**
Use **fresh platelets** from healthy donors or stored platelets (0.1% sodium azide). - collect blood in citrate-containing tubes using a 21-gauge needle (avoid vacuum suction), from donors with blood group O and known to express the HPA-1a antigen - centrifuge whole blood for obtaining PRP (200× *g*, 10 min) - wash platelets twice with phosphate-buffered saline containing BSA 0.1%.
**III. Test Methods**
**Drug preparation**: Dissolve each drug to be tested in adequate solution, according to its solubility. The suspected drug should be tested at therapeutic concentration (i.e., 0.3 mg/mL for vancomycin) **Flow cytometry and Enzyme immunoassays (EIAs)** can be used for detecting DDabs. In both assays, healthy donor platelets are incubated with patient serum or plasma in the presence and absence of drug. When required, the drug must be present in all buffers and during all steps, including washings. After washings, platelet-associated DDabs will be detected using fluorescent-labeled anti-human IgG and IgM (flow cytometry), or using an enzyme-labeled goat anti-Human IgG and IgM (EIA).
**IV. Patient Samples and Controls**
Patient samples and negative/positive controls (usually serum) must always be tested. **Positive control**: serum or plasma sample from one previous patient with DITP, or with anti-HPA1 antibody (WHO standard 106/05 or patient sample), but test HPA1 positive platelets. **Negative control**: serum or plasma sample from patient treated with the drug and normal platelet count, or from healthy control.

BSA: bovine serum albumin, DDabs: drug-dependent antibodies, EDTA: Ethylenediaminetetraacetic acid, EIA: enzyme immunoassay, HPA: human platelet antigen, ISTH: International Society on Thrombosis and Haemostasis, PRP: platelet-rich plasma, SSC: Scientific and Standardization Committee, WHO: world health organization.
